# Safety of indwelling pleural catheter use in patients undergoing chemotherapy: a five-year retrospective evaluation

**DOI:** 10.1186/s12890-016-0203-7

**Published:** 2016-03-11

**Authors:** Charleen Chan Wah Hak, Parthipan Sivakumar, Liju Ahmed

**Affiliations:** Respiratory Medicine, Guy’s and St Thomas’ NHS Foundation Trust, London, UK; School of Medicine, King’s College London, London, UK

**Keywords:** Antineoplastic agents, Catheters, Indwelling, Pleura, Pleural effusion, Malignant

## Abstract

**Background:**

Indwelling pleural catheters (IPC) are increasingly becoming a first-line treatment in the management of malignant pleural effusions. Ambulatory management using IPC are increasingly used in this patient group whilst they are receiving concurrent chemotherapy. There are currently no prospective trials examining IPC safety in chemotherapy. This study’s objective is to determine if IPC insertion is safe in patients undergoing chemotherapy.

**Methods:**

We conducted a retrospective analysis of all patients who underwent IPC insertion for malignant pleural effusion at our trust from September 2010 to December 2014. Data was collected on IPC insertion and removal, tumour type, systemic chemotherapy, pleural infection and other complications.

**Results:**

One hundred four patients were identified, 43 in chemotherapy group and 61 in non-chemotherapy group. The incidence of pleural infection in chemotherapy group *vs* non-chemotherapy group, 4 (9.3 %) *vs* 3 (4.9 %) respectively, was not statistically different (Fisher’s exact *p* = 0.311). There was no significant difference in six-month infection-free duration from the date of IPC insertion (log rank *p* = 0.394*).* Overall six-month mortality in chemotherapy group was significantly lower than in non-chemotherapy group (log rank *p* = 0.007).

**Conclusions:**

This is the second largest retrospective case–control series that concludes systemic chemotherapy is safe in patients with IPC undergoing chemotherapy.

## Background

Malignant pleural effusions (MPE) are a common presentation in the advanced and disseminated stages of malignancy, complicating nearly 50 % of all lung and breast cancers [1]. Extrapolated data estimates 50 000 new cases of MPE per year in the UK, translating to one new case per 1000 population per year [2]. The development of MPE can result in significant morbidity including disabling breathlessness, pain and reduced physical capability. Treatment is aimed at relieving breathlessness and improving quality of life. MPE is associated with a poor prognosis, dependent on the type and stage of cancer, with a median survival of only 74 days in lung cancer and less than 50 days in urological cancer, sarcoma and melanoma groups [3]. Therefore a definitive single pleural intervention is important in a patient group with a poor prognosis.

If the primary tumour type is particularly sensitive to chemotherapy, oncological treatment may result in regression of the effusion and symptom alleviation. However, the initial presentation of malignancy may be breathlessness secondary to MPE, which requires pleural intervention prior to starting chemotherapy.

There are two main therapeutic strategies to palliate recurrent MPE. The most common strategy is to attempt pleurodesis either via chest drain or by thoracoscopy. The alternative is continuous drainage with an indwelling pleural catheter (IPC). Superiority of one method over the other is not clear [4–6].

The popularity and use of IPC has grown over the last decade as they permit outpatient ambulatory management of MPE with low complication rates [7–10]. Although conventionally reserved for patients with trapped lung or previous failed pleurodesis, more recently a novel pathway of administering talc slurry via the IPC has been shown to be safe and efficacious [9]. Trials evaluating IPC as a first line treatment strategy are currently underway, including the IPC PLUS trial (UKCRN 73255764) investigating the success rate of talc pleurodesis via IPC and the OPTIMUM trial (UKCRN 19615) examining health-related quality of life outcomes.

There is an associated risk of infection with the semi-permanent nature of IPC, ranging between 2.2 and 12 % [11, 12]. In current literature, there are no randomised controlled trials comparing infection rate in patients with IPC to other pleural interventions and no prospective trials primarily examining IPC safety in chemotherapy. Of the published case series available, there is reportedly no increased risk of infection in patients with IPC undergoing chemotherapy [13, 14]. Our primary study objective is to determine the safety of IPC insertion and longer-term use in chemotherapy.

## Methods

### Study design

This study is a retrospective case–control series. IPC have been adopted as part of first-line management pathway for malignant effusions at Guy’s and St Thomas’ NHS Foundation Trust, London, UK, since 2010. We performed a review of hospital inpatient documentation, clinic letters, microbiological and biochemical results. Data collection was conducted over the period of April to July 2015, on date of IPC insertion and removal (if known), tumour type, chemotherapy, six-month incidence of pleural infection and other complication rates, as well as six-month mortality from date of IPC insertion.

Following consultation with our local Research and Development department at Guy’s and St Thomas’ NHS Foundation Trust, we obtained local clinical governance committee approval for the use of patient records. As a retrospective service evaluation, written patient informed consent and regional ethics approval was not required. Patient identifiable information was not recorded to maintain patient confidentiality.

### Participants

We included all patients with symptomatic MPE who underwent IPC insertion between September 2010 and December 2014. Patients with benign disease were excluded.

### Interventions

All procedures were performed on an outpatient basis, unless the patient had already been admitted to hospital. We defined chemotherapy intervention as systemic cytotoxic, molecular or biological therapy, excluding hormonal therapy.

### Assessments

The primary endpoint of this study was pleural infection whilst an IPC was in-situ. Pleural infection was defined as, 1) A clinical presentation compatible with pleural infection requiring antibiotic treatment, and 2) A pleural fluid sample that meets at least one or more of the following criteria:

- Purulent
- Gram stain positive for bacteria
- Bacterial culture positive.

Secondary endpoints were defined as IPC-related cellulitis, pain and drain blockage and six-month mortality data from the date of IPC insertion.

Statistical analyses were performed using IBM SPSS version 22. Chi-squared (χ2) or Fishers exact tests (where indicated) were performed on dichotomous categorical variables. Log-rank test was used to compare infection-free survival as well as overall survival between chemotherapy and non-chemotherapy groups. Survival distributions were depicted by Kaplan-Meier survival curves.

## Results

One hundred four patients underwent IPC insertion for MPE between September 2010 and December 2014, 43 in chemotherapy group and 61 in non-chemotherapy group (Table 1).

**Table 1:** Patient demographics and interventions

	Chemotherapy	No chemotherapy
No. of patients	43 (40 %)	61 (60 %)
Mean age (yrs) (SD)	64 (15)	68 (12)
Cancer primary		
Lung (Small cell)	0 (0 %)	1 (2 %)
Lung (Non-small cell)	17 (40 %)	16 (26 %)
Mesothelioma	3 (7 %)	9 (15 %)
Breast	11 (26 %)	16 (26 %)
Other	12 (28 %)	19 (31 %)
Median duration IPC in-situ (days) (IQR)	28 (14–58)	69 (47–143)
Median duration of concurrent chemotherapy (IQR)	52 (21–105) (~4 cycles)	-

**Fig. 1:**
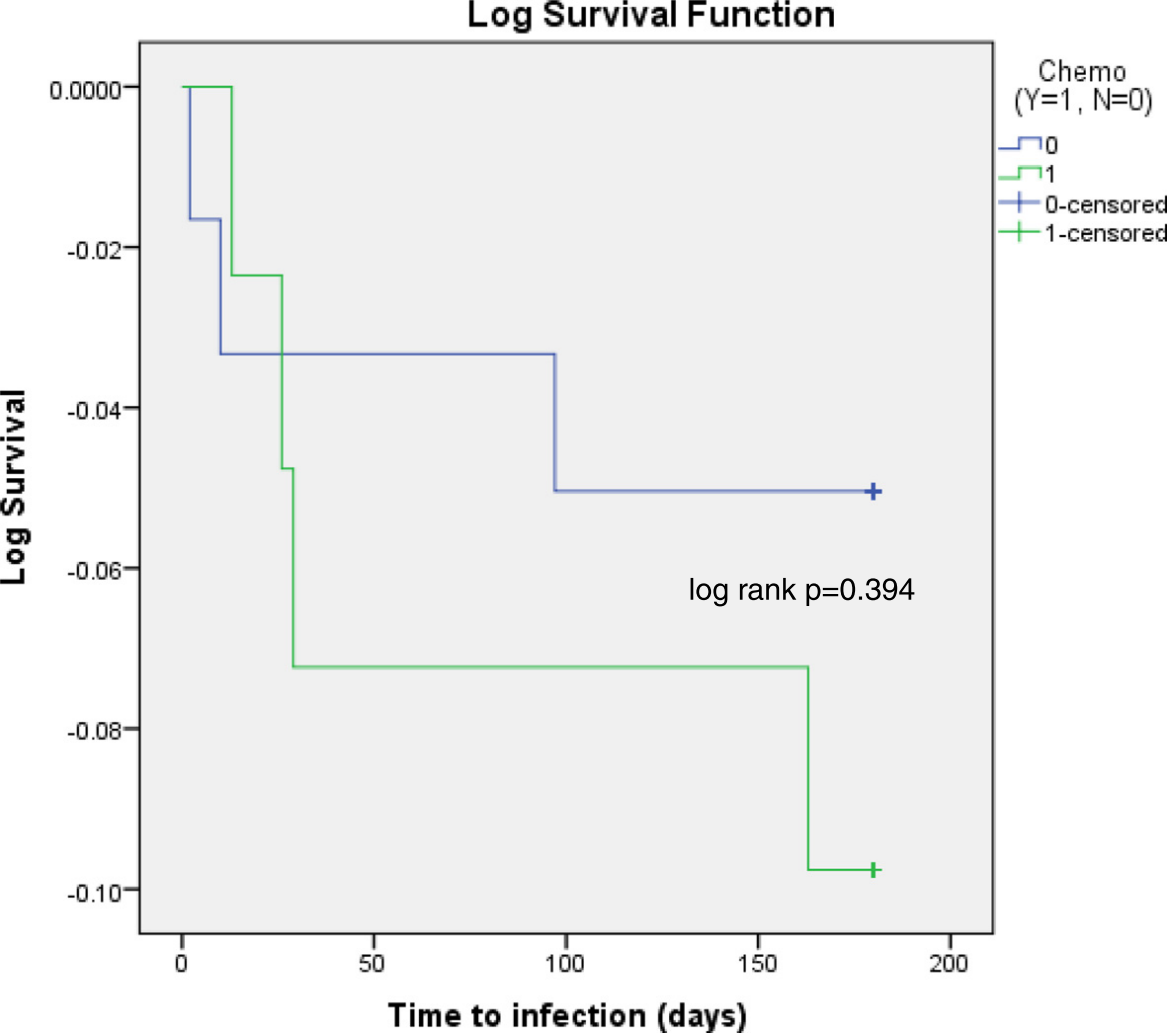
Time to pleural infection over six-months between chemotherapy and non-chemotherapy groups (Kaplan-Meier). Event: pleural infection. *Blue line*: non-chemotherapy group; *Green line*: chemotherapy group

The incidence of pleural infection at six-month follow-up in chemotherapy group *vs* non-chemotherapy group was 4 (9.3 %) *vs* 3 (4.9 %) respectively. This was not statistically different (Fisher’s exact *p* = 0.311) (Table 2). There was no significant difference in the post-insertion time to infection between both groups over six-month follow-up (log rank *p* = 0.394), where the occurrence of pleural infection is considered an “event” in the Kaplan-Meier curve (Fig. 1).

**Table 2:** Complications and mortality

Outcome	Chemotherapy	Non-chemotherapy	Statistical significance
Complications			
Pleural infection	4 (9.3 %)	3 (4.9 %)	*p* = 0.311
Cellulitis	2 (4.7 %)	1 (1.6 %)	*p* = 0.370
Pain	2 (4.7 %)	2 (3.3 %)	*p* = 0.550
Drain blockage	1 (2.3 %)	0 (0 %)	*p* = 0.413
6-month mortality	15 (35 %)	36 (59 %)	*p* = 0.007

In the seven patients identified with pleural infection (Table 3), three patients had diagnoses of empyema that led to drain removal, and two patients (^a^) had pleural infection diagnosed within two months of date of death. Organisms isolated in pleural fluid include: *Staphylococcus epidermidis* (CoNS), *Staphylococcus lugdunesis*, *Staphylococcus aureus*, *Pseudomonas* and *Enterococcus faecalis*. Only two patients out of 43 in the chemotherapy group had documented neutropenic sepsis. In one patient, this occurred after the IPC was removed, and the second patient was hospitalised with neutropenic sepsis secondary to pleural infection, was palliated and died during the same admission.

**Table 3:** Pleural infection details

Patient	Chemotherapy	a. Purulent	b. Gram stain positive	c. Bacterial culture positive	Organisms isolated	IPC removed due to empyema
1^a^	No	Yes	Yes	Yes	*Staphylococcus lugdunesis*	Yes
2	No	No	No	No	-	Yes
3	No	Yes	Yes	No	-	No
4	Yes	No	No	Yes	*Staphylococcus epidermidis* (CoNS)	No
5	Yes	No	Yes	Yes	*Staphylococcus epidermidis* (CoNS)	No
6^a^	Yes	No	No	Yes	*Staphylococcus aureus*	No
7	Yes	Yes	Yes	Yes	*Pseudomonas, Enterococcus faecalis*	Yes

^a^Patients with pleural infection diagnosed within two months of date of death

The incidence of other complications between chemotherapy and non-chemotherapy groups were not statistically significant: cellulitis (*p* = 0.370); pain (*p* = 0.550); drain blockage (*p* = 0.413) (Table 2).

Within the chemotherapy group, each patient underwent complex individualised regimens often with multiple chemotherapy types combined or sequentially (Table 4). As such, no clear association can be made between type of chemotherapy used and incidence of pleural infection. Of the four pleural infections in the chemotherapy group, four different regimes were used, 1: vinorelbine followed by paclitaxel, 2: topetcan followed by gemcitabine, 3: carboplatin and paclitaxel, and 4: capcetabine and lapatinib.

**Table 4:** Chemotherapy types

Chemotherapy type^a^	Patient number	Associated pleural infection
Antimetabolites	24	2
Platins	22	1
Taxanes	10	2
EGFR/TKI inhibitors	8	1
Biologics	5	0
Topoisomerase inhibitors	1	1
Vinca alkaloids	1	1
Anthracyclines	1	0

^a^Chemotherapy type counted individually if part of multi-agent regime in the same patient

**Fig. 2:**
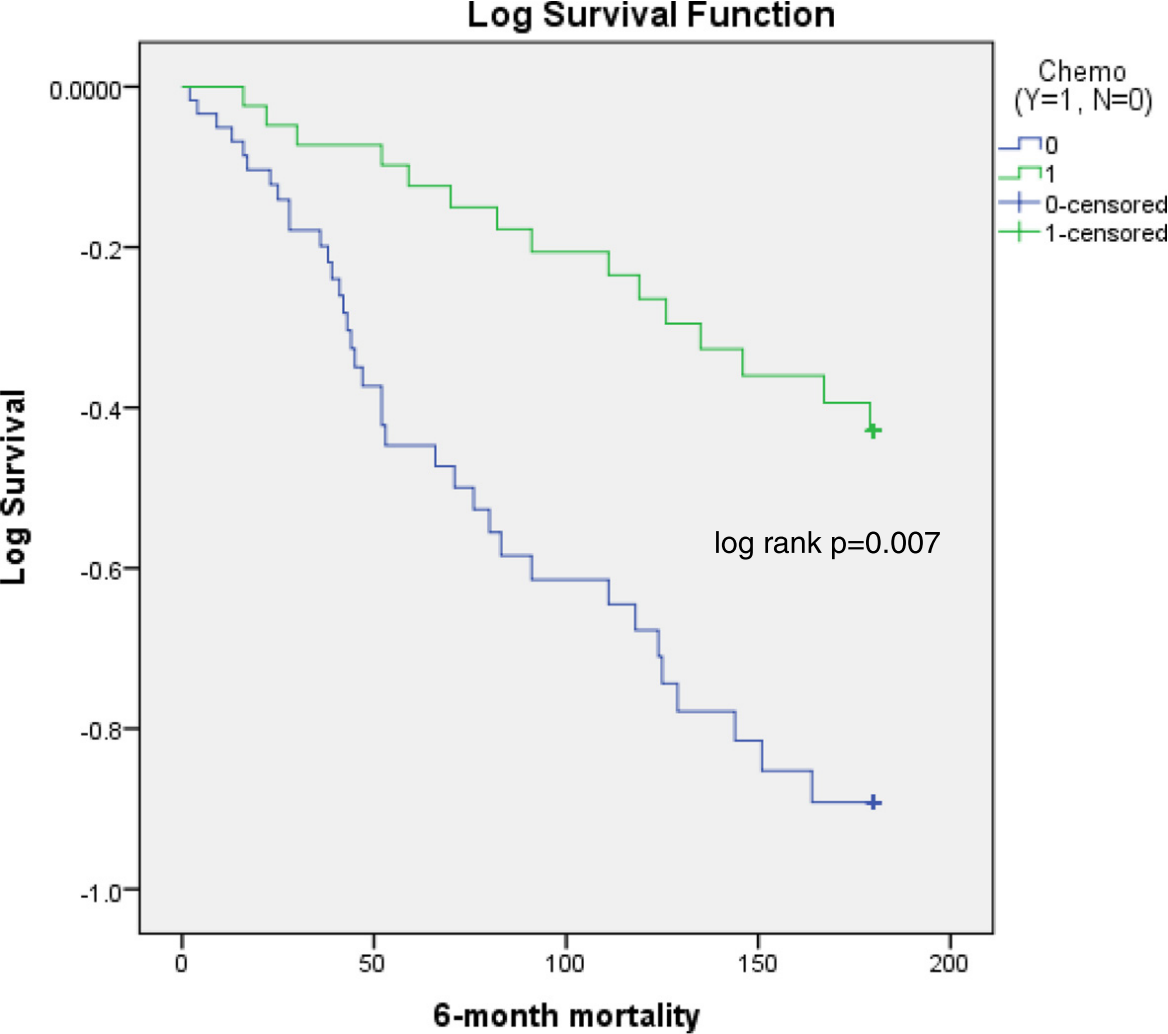
Six-month mortality data between chemotherapy and non-chemotherapy groups (Kaplan-Meier). Event: patient death. *Blue line*: non-chemotherapy group; *Green line*: chemotherapy group

Overall six-month mortality in chemotherapy group (*n* = 15) was significantly lower than in non-chemotherapy group (*n* = 36), 35 % *vs* 59 % respectively (log rank *p* = 0.007) (Fig. 2).

## Discussion

IPC are increasingly used to manage malignant pleural effusions in a heterogeneous population, including those receiving chemotherapy. The complication rate of these devices is low with no significant difference in adverse events with IPC compared to chest drain insertion and talc pleurodesis [4].

Our retrospective data shows that there is no increased rate of pleural or skin infection in patients undergoing chemotherapy compared to the non-chemotherapy group. These results are supported by the limited literature available. The largest case series by Mekhaeil et al. comprised of 262 patients with a reported pleural infection rate of 5.2 % in the chemotherapy group, which was not significantly different from the non-chemotherapy group [13]. Morel et al. found similar results [14]. In the TIME2 study of five serious pleural infections in the IPC group, only one patient received systemic chemotherapy at the time [4]. The significant survival benefit found in the chemotherapy group is reassuring. In our population, 32.7 % of patients survived less than three months and 12.5 % survived less than one month from the date IPC insertion. Similarly, a third of patients recruited into the TIME2 study died within three months (despite an exclusion criteria for trial entry of predicted survival of < three months) [4]. Although clinical trials investigating MPE aim to recruit patients with a reasonable prognosis, in practice, IPC are not being exclusively used in this patient group.

There is a lack of consensus on a “safe” window for IPC insertion, particularly in the context of chemotherapy. In our practice, we consider neutropenia, thrombocytopaenia or irreversible anticoagulation or coagulopathy as contraindications to IPC implantation, and would delay IPC insertion until these have recovered. With increasingly complex chemotherapy regimes and targeted therapies, close liaison with oncologists is vital for a positive patient outcome.

The main limitation of this study is that given adverse events are a rare occurrence, our study may not be sufficiently powered to detect a difference. The relationship between chemotherapeutic agent and incidence of pleural infection was not our primary endpoint and requires further evaluation. Although a retrospective analysis, our open inclusion criteria should prevent any bias that may affect the selection of controls. The strength of our data is in its application in everyday practice.

There is a clear need for further prospective study to examine the safety of IPC insertion and longer-term use in patients with MPE undergoing chemotherapy. Important prospective evidence as secondary outcomes are likely to be obtained from the IPC PLUS (UKCRN 73255764) and the OPTIMUM trials (UKCRN 19615), which should provide the data needed. Given the increasing use of IPC worldwide, international collaboration with a prospective database will allow the pooling of long-term outcomes.

## Conclusions

This is the second largest retrospective case–control series that advocates the safety of IPC insertion as a first-line treatment for malignant effusion. Our data suggests that patients undergoing chemotherapy should not be denied ambulatory management of their effusion with an IPC.

### Ethics approval and consent to participate

As a retrospective service evaluation, written patient informed consent and regional ethics approval was not required. Consultation with the local Research and Development department at Guy’s and St Thomas’ NHS Foundation Trust clarified that local clinical governance committee approval was needed for the use of patient records, which was obtained.

### Consent for publication

Not applicable.

### Availability of data and materials

Due to our local governance policy, we do not have permission to make the data sets on which the conclusions of the paper rely publicly available. A truncated data set (removing all potentially identifying features) may be made available on an individual request basis.

## Abbreviations

IPC: indwelling pleural catheter(s)
MPE: malignant pleural effusion(s)
